# On the recurrence rate of cutaneous tumors treated exclusively by micrographic surgery^☆^

**DOI:** 10.1016/j.abd.2022.12.001

**Published:** 2023-02-17

**Authors:** Luiz Eduardo Fabrício de Melo Garbers, Ana Carolina Miola, Luis Fernando Figueiredo Kopke, Hélio Amante Miot

**Affiliations:** a Department of Dermatology, Faculty of Medicine, Universidade Estadual Paulista, Botucatu, SP, Brazil; b Department of Dermatology, Hospital Universitário, Universidade Federal de Santa Catarina, Florianópolis, SC, Brazil

Dear Editor,

We read with interest the article by Dr. Otsuka et al., in which they used a peripheral sampling technique with a parallel incision of the lateral and deep surgical margins of cutaneous carcinomas (basal cell and squamous cell carcinomas). Moreover, they validated the concordance of the identification of compromised margins with subsequent analysis of the paraffin-embedded specimens.^1^ We would like to congratulate the authors and make comments regarding the method, its validation, and conclusions about the recurrence rate.

We encourage the study of operative techniques in micrographic surgery, as well as in the processing of surgical specimens, which may lead to effective and faster procedures, with lower cost and morbidity rates. Initially, for clarifying purposes, it should be noted that the technique used by the authors is identical to the Tübingen method, one of the most employed modalities in Europe.^2^

When meta-analytically analyzed, the results of 10,424 basal cell carcinomas operated on by micrographic surgery, the different micrographic surgery techniques do not show overall differences in recurrence rates between them (1% to 3%), although there are no parallel comparative studies that could explore their peculiarities. Primary tumors have a recurrence rate that ranges from 1% to 3%, and relapses, of 2% to 5%.^3^, ^4^

Interestingly, false-negative margins can occur in discontinuous tumors (such as multicentric basal cell carcinoma - BCCs), with thin areas (e.g., tumors with perineural invasion), recurrences under flaps/grafts, and because of technical problems during preparation of the slides. On the other hand, false-positive margins can occur due to confusion with cross-sections of follicular bulbs, due to the inflammatory infiltrate, or in the initial spare sectionning of specimens in the cryostat, necessary for preparing the slides. For this reason, validation of the coincidence of surgical margins in paraffin, as used in the work by Otsuka et al., may not be plausible, as it may overestimate the involvement of the margins, since the outermost sections were previously sampled and analyzed intraoperatively, in thicker sections than the ones in paraffin.

When analyzing, separately the recurrence rates described by the authors for primary basal cell carcinomas (0.3%) and the recurrent ones (4.3%), we found that there was supplementation of the surgical treatment with radiotherapy in 97% of the cases, which is unusual, especially for basal cell carcinomas (80% of the sample), given the use of a surgical technique that verifies 100% of the surgical margins aiming at complete cure and recurrence prevention. Although the literature has publications that suggest exclusive radiotherapy as a treatment option for BCCs, with good results and local control of up to 96% of cases, studies on the effectiveness of adjuvant radiotherapy in preventing recurrences in micrographic surgeries are still necessary.^5^ In the meantime, the recurrence rate found by the authors cannot be attributed only to the surgical technique, but to the combination between micrographic surgery and complementary radiotherapy.

Finally, the completion of micrographic surgeries with only one stage in 72% of cases prompts the discussion of indication criteria, aiming to maximize the cost-benefit to the detriment of conventional oncological surgery, since the latter is more accessible, both technically and financially for the health system.

## Financial support

None declared.

## Authors’ contributions

Luiz Eduardo Fabrício de Melo Garbers: Design of the study; writing of the manuscript; review and approval of the final version of the manuscript.

Ana Carolina Miola: Design of the study; writing of the manuscript; review and approval of the final version of the manuscript.

Luis Fernando Figueiredo Kopke: Design of the study; writing of the manuscript; review and approval of the final version of the manuscript.

Hélio Amante Miot: Design of the study; writing of the manuscript; review and approval of the final version of the manuscript.

## Conflicts of interest

None declared.
